# Trends in adults’ energy imbalance gaps over two decades in Belgium using system dynamics modelling

**DOI:** 10.1186/s40795-023-00721-0

**Published:** 2023-05-27

**Authors:** Saeideh Fallah-Fini, Tannaz Rezaei, Karin De Ridder, Stefanie Vandevijvere

**Affiliations:** 1 Industrial and Manufacturing Engineering Department, California State Polytechnic University, Pomona, CA USA; 2 Computer Science Department, California State Polytechnic University, Pomona, CA USA; 3 Sciensano, Department of Epidemiology and Public health, J.Wytsmanstraat 14, Brussels, 1050 Belgium

**Keywords:** Obesity, System dynamics modelling, Belgium, Health interview surveys, Adults

## Abstract

**Background:**

The energy imbalance gap (EIG) represents the average daily difference between energy intake and energy expenditure. The maintenance energy gap (MEG) captures the increased energy intake needed to maintain a higher average bodyweight compared with an initial distribution of bodyweight. This study quantified the dynamics of the EIG and MEG over time and across different genders/regions/BMI groups for Belgian adults.

**Methods:**

A validated system dynamics model was adapted to estimate the trends/dynamics of the EIG among different subpopulations over two decades in Belgium. The model was calibrated using data from the six Belgian national Health Interview Surveys (1997, 2001, 2004, 2008, 2013, 2018).

**Results:**

EIG was negative for all BMI groups among Belgian females in 2018, implying the start of a decrease in prevalence of overweight/obesity in this subpopulation. However, this was not the case among Belgian males. Flemish and Walloon males had positive EIGs across BMI groups in 2018, however, Brussels’ males showed negative EIGs across BMI groups. Flemish and Brussels’ females showed negative EIGs across all BMI groups in 2018, while Walloon females showed positive EIGs across almost all BMI groups. According to the MEG, Belgian men consumed (and expended) on average 59 kcal/day more in 2018 than in 1997 to maintain their heavier body weight. The MEG for Belgian women was 46 kcal/day in 2018, triple the MEG in 2004.

**Conclusions:**

The detailed heterogeneous trends of the EIG describe the obesity patterns for different subpopulations in Belgium and could be used to model the differential effects of specific nutrition policies targeting energy intake.

**Supplementary Information:**

The online version contains supplementary material available at 10.1186/s40795-023-00721-0.

## Background

The global prevalence of obesity has increased considerably over the past 40 years, from 3 to 11% among men and from 6 to 15% among women since 1975 [1]. Most European countries are currently in stage 3 of the so called ‘obesity transition’, whereby the prevalence of overweight and obesity among those with lower socioeconomic status surpasses that of those with higher socioeconomic status, and plateaus in prevalence can be observed in women with high socioeconomic status and in children for some countries [2]. The latest Health Interview Survey (HIS) in Belgium in 2018 showed that almost half (49.3%) of the Belgian adult population was overweight while 15.9% suffered from obesity [3]. The prevalence of both overweight and obesity significantly increased since the first HIS in 1997 (41.3% and 10.8%, respectively). As in other European countries, both overweight and obesity are associated with education level: lower educated people have a higher risk of overweight and obesity [2]. In Belgium, among the lowest educated population group, almost two out of three (61.8%) adults are overweight and about a quarter (21.8%) suffers from obesity [3].

Generally, in Belgium, there has never been a comprehensive strategy in place to combat and prevent overweight and obesity and their related inequalities. The World Health Organization (WHO) recommends governments to implement comprehensive population-wide policies (e.g., restricting marketing for unhealthy foods to children, introducing taxes on unhealthy foods, policies on healthy foods in schools) to tackle overweight and obesity [4]. During the period 2005–2010 there was a national nutrition and health plan for Belgium, which mainly focused on improving breastfeeding rates, improving micronutrient status of different population groups and stimulating voluntary actions by the food industry to reduce population salt intakes [5].

Based on the Belgian Food Consumption Survey 2014, the usual proportion of daily energy intake from ultra-processed food and drink products in Belgium was 33.3% for children, 29.2% for adolescents and 29.6% for adults. There were no significant changes in relation to this indicator compared to the previous, similar representative survey in 2004 [6]. While a second nutrition and health plan has not been introduced since 2010, some isolated voluntary actions have since been introduced by the Minister of Health, such as the Convenant Balanced Diet [7] in 2012 and the voluntary Nutri-Score front-of-pack label [8] in 2019. Within the Convenant, the food industry committed to reduce population energy intake by 5% through reducing sugar and saturated fat in their products, between 2012 and 2017 [7].

Obesity is the result of the imbalance between energy intake and energy expenditure, quantified by measures such as the energy imbalance gap (EIG) and the maintenance energy gap (MEG). The EIG represents the average daily difference between energy intake and expenditure and is the driver behind changes in body mass [9]. The MEG captures the increased energy intake needed to maintain higher average bodyweight compared with an initial (e.g., the late 1990s) distribution of bodyweight [9]. Analyzing the dynamics of EIG and MEG will allow for better understanding and design of public health policies, estimating the effect of public health interventions, and estimating the contribution of different drivers of obesity [9–13]. Except a limited number of studies [9, 10, 14], most of the estimates of the EIG and MEG in the literature are based on simple models aggregated over a long period of time for the entire population [12, 13, 15].

In this study, we adapted a novel method developed in system dynamics [16], previously applied in the United States [9], New Zealand [10], and Japan [14], to estimate and understand the dynamics and trends in the energy imbalance gaps (EIG) and energy maintenance gaps (MEG) among different Belgian adult subpopulations and regions over the past two decades.

## Methods

We used a novel method developed in system dynamics [16] to connect the micro-level dynamics of BMI of individuals to the macro-level dynamics of the distribution of BMI in the population. Since, based on the Belgian Health Interview Surveys [3], overweight and obesity trends were different for male and female adults (aged 20 to 74 years old) and across the major regions, we divided the Belgium population, first, into two subpopulations based on their gender (male and female) and then into six subpopulations based on their major region (Flanders, Wallonia, Brussels) and gender. We estimated the trends of EIG and MEG for each subpopulation.

For each subpopulation, we first divided the range of possible values of BMI into 14 different partitions or classes (e.g., (15,18], (18,20], (20,23], …., [65,68]), each of which represented a distinct stock. Each stock contained the part of the population whose BMI fell within the BMI interval associated with that stock.

We assigned a hypothetical representative individual to each stock, where the BMI of the representative individual was the average of the BMI interval associated with that stock.

We modeled the dynamics of body weight gain and loss of each representative individual using the Hall et al. model [11] of adult metabolism and body weight dynamics. Weight gain/loss of representative individuals was modeled as the result of imbalance between their energy intake and energy expenditure, represented by their EIG.

As the representative individual associated with each BMI class (stock) gained/lost weight, he/she pushed the population of that stock into the neighboring BMI classes, thus, causing a shift in the distribution of BMI over time. The rate of change of the BMI of representative individuals provided the speed by which population BMI distribution shifted to the right or left.

For each subpopulation *j*, the EIG of the representative individual associated with each BMI class *k* (*k = 1, …, 14*) at any time *t* (*t* = 1997, …, 2018) was modeled as a function of their equilibrium energy expenditure (*EE*_*jk*_) and energy gap multiplier (${\mu }_{jkt}$), as shown in Eq. (1). The equilibrium energy expenditure of each representative individual associated with BMI class *k* shows the energy required for normal activity and maintenance of the body weight.

1 $$EIG_{jkt}=EI_{jkt}-EE_{jk}=\mu_{jkt}\;\ast\;EE_{jk}$$

We then calculated the energy intake of the representative individual of BMI class *k* (${EI}_{jkt}$) by adding the energy imbalance gap ${EIG}_{jkt}$ to the equilibrium energy expenditure ${EE}_{jk}$. We modeled the energy imbalance gap ${\mu }_{jkt}$ as a function of time, BMI class, and interaction of them as shown in Eq. (2). We specified general models to allow for flexible and non-linear relationships between time and BMI in the model.

2 $$\begin{array}{c}\mu_{jkt}=BMI\;Effect_{jk}+Time\;Effect_j+Interaction\;of\;Time\;and\;BMi\;Effects_{jk}\\BMI\;Effect_{jk}=\beta_{1j+}\beta_2BMI_{jk}+\beta_{3j}\left(BMI_{jk}\right)^{\beta_{4j}}\\Time\;Effect_j=\beta_{5j}Time+\beta_{6j}\left(Time\right)^2\beta_{7j}\left(Time\right)^3\\Interaction\;Effect_{jk}=\beta_{8j}BMI_{jk}Time\end{array}$$

To make sure the system dynamics model was demographically representative for the Belgium adult population, we also modeled the death rate and the rate of transition from childhood (19-year-old individuals) into adulthood for each BMI class. To take into account the differential mortality attributable to very low or high BMI, we used the mortality adjustment curves developed by Gray [17].

Note that there are different causal pathways discussed in obesity models in the literature [18]. One causal pathway which also was used in this paper is based on the first law of thermodynamics and states that for adipose tissue fat storage to increase, there should be a positive energy imbalance gap [11, 19]. In this view, which is widely accepted in the literature, positive energy imbalance gap is upstream (cause) of increase in fat mass. The other causal pathway is the carbohydrate-insulin model [18, 20, 21]. In this model the hormonal response to highly processed carbohydrates leads to further deposition of energy in adipose tissue. The increasing adiposity then drives overeating. In the carbohydrate-insulin model positive energy imbalance is downstream to adipose tissue fat storage [18]. The Hall et al. model [11] of adult metabolism that was used in our model to capture body weight dynamics of representative individuals adopted the first view. Thus, the results, discussion, and conclusion provided in the rest of this paper is based on the first view on causal pathways of obesity.

### Calibration

We used the cross-sectional data from the six Belgian Health Interview Surveys from 1997, 2001, 2004, 2008, 2013 and 2018 to obtain population level distribution of BMI for age 20 to 74 years for different subpopulations in this study. The first edition of the HIS was conducted in 1997 for the general population. Since then it has been repeated periodically over time up to its 6th edition in 2018. One of the main objectives of the HIS is to measure the health status of the population in Belgium, accounting also for the three regional sub-populations (in Flanders, Wallonia and Brussels-Capital). Based on sample size calculations, the total number of successful participants for the basic sample is generally set to 10,000 (3500 for Flanders, 3500 for Wallonia, 300 for East Belgium and 3000 for Brussels). To select the sample, a stratified clustered multi-stage design was used. More details on the design and sampling of the survey can be found elsewhere [22]. Data collection in the HIS takes place using two standardized questionnaires: (1) a questionnaire administered in a face-to-face interview setting, and (2) a paper questionnaire handed out to participants for self-completion. Height and weight of participants is self-reported and collected through the face-to-face questionnaire. Data of transition rates from adolescence (19 years old) to adulthood (age 20 to 74) were also obtained from the HIS. All HIS surveys have been approved by the Data Protection Authority in Belgium and by the Ethics Committee of Ghent University Hospital. Participants provided written informed consent. The data for all-cause mortality was obtained from the Belgian Mortality Monitoring (Be-Momo) project [23].

For each subpopulation, we initialized the model using the BMI distribution data obtained from the first HIS (i.e., 1997). The model was then simulated through 2018. We used the maximum likelihood method to estimate the beta parameters of EIG (and consequently estimate the EIG) so that the distribution of BMI generated by the model was as close as possible to the distribution of BMI obtained from the HIS in all years for which data was available (i.e., 1997, 2001, 2004, 2008, 2013, 2018). The overall log-likelihood function summed up the logarithm of likelihood values across survey years.

We used a non-linear optimization method to find the beta parameters of EIG so that the overall log-likelihood function was maximized.

The estimated beta parameters are reported in Table 1. We also calculated the MEG for each subpopulation to represent the increase in energy intake needed to maintain the higher average body weight comparted to the one in 1997.

**Table 1:** Model Parameters Estimated through Calibration for all Subpopulations

		BMI Effect				Time Effect			Interaction Effect
		${\varvec{\beta }}_{1}$	${\varvec{\beta }}_{2}$	${\varvec{\beta }}_{3}$	${\varvec{\beta }}_{4}$	${\varvec{\beta }}_{5}$	${\varvec{\beta }}_{6}$	${\varvec{\beta }}_{7}$	${\varvec{\beta }}_{8}$
**Belgium**	**Males**	-0.363	0.079	0.306	-1.00	-0.014	0.024	-0.009	-0.004
	**Females**	-0.192	0.044	0.174	-0.068	-0.062	0.158	-0.101	-0.017
**Flemish region**	**Males**	0.130	0.083	-0.206	0.264	0.013	-0.100	0.080	0.036
	**Females**	-0.207	0.084	0.160	-0.172	-0.117	0.291	-0.189	-0.015
**Brussels**	**Males**	-0.251	0.120	0.187	-0.155	-0.053	0.201	-0.127	-0.088
	**Females**	-0.365	0.060	0.337	-0.060	-0.137	0.338	-0.219	-0.010
**Wallonia**	**Males**	-0.355	0.121	0.266	-0.172	-0.018	0.055	-0.037	-0.008
	**Females**	0.137	-0.014	-0.117	-0.063	-0.009	-0.025	0.031	0.005

To validate our results, we used the one-sample Kolmogorov–Smirnov Goodness of Fit test to examine whether the BMI distribution simulated by the system dynamics model was different from the BMI distribution observed in the surveys. Table 2 shows the Kolmogorov–Smirnov test results. We conducted the data processing by Stata version 14 (StataCorp, College Station, TX, USA) and all simulations and optimizations by Vensim™ (Ventana Systems, Inc., Harvard, MA, USA).

**Table 2:** K-S Test Goodness of Fit Results for all Subpopulations in Belgium

			1997	2001	2004	2008	2013	2018
Belgium	Males	critical value	0.023	0.021	0.021	0.024	0.023	0.022
		KS Test Statistics	0.004	0.014	0.010	0.014	0.021	0.008
	Femals	critical value	0.023	0.021	0.021	0.023	0.022	0.021
		KS Test Statistics	0.014	0.011	0.011	0.014	0.009	0.010
Flemish region	Males	critical value	0.038	0.036	0.035	0.039	0.040	0.036
		KS Test Statistics	0.010	0.026	0.019	0.015	0.025	0.007
	Femals	critical value	0.039	0.036	0.035	0.039	0.039	0.035
		KS Test Statistics	0.018	0.030	0.012	0.034	0.010	0.016
Brussels	Males	critical value	0.042	0.044	0.043	0.044	0.043	0.043
		KS Test Statistics	0.034	0.010	0.015	0.020	0.013	0.022
	Femals	critical value	0.041	0.042	0.041	0.042	0.041	0.041
		KS Test Statistics	0.015	0.018	0.013	0.020	0.016	0.019
Wallonia	Males	critical value	0.039	0.034	0.035	0.040	0.036	0.036
		KS Test Statistics	0.006	0.013	0.007	0.011	0.021	0.016
	Femals	critical value	0.038	0.033	0.034	0.039	0.035	0.036
		KS Test Statistics	0.023	0.012	0.008	0.030	0.017	0.011

## Results

**Fig. 1:**
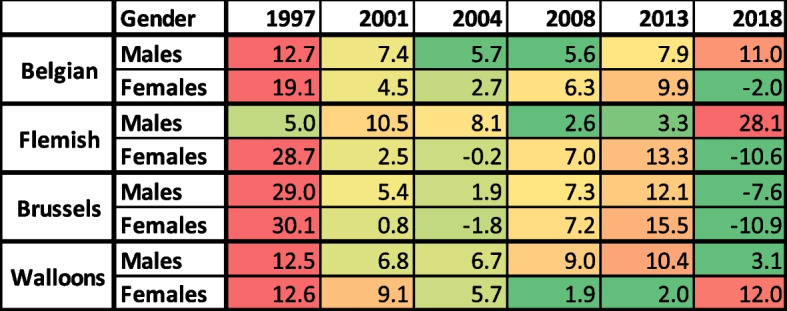
Estimated overall energy imbalance gap (EIG) in kilocalories per day for Belgian adults by gender and region for survey years. Estimated overall EIG across all years is reported in Supporting Information Figures S1

**Fig. 2:**
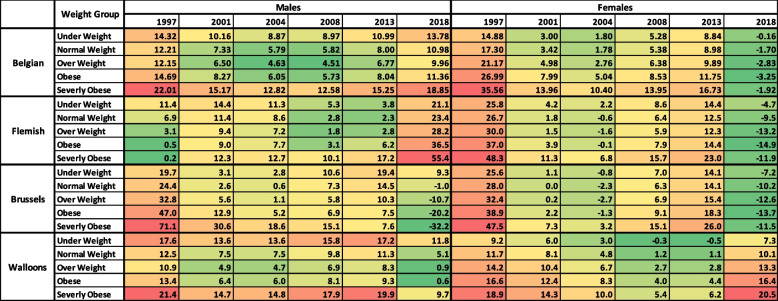
Estimated energy imbalance gap (EIG) in kilocalories per day for Belgian adults (age 20–74 years) by gender, region, and weight group in Belgium for survey years. Estimated EIG across all years is reported in Supporting Information Figures S2

Positive values for the EIG indicate gaining weight on average, the negative values indicate losing weight, and zero values reflect no change in BMI. In the heat maps shown in Figs. 1 and 2, the green colour shows relatively small values for the estimated EIG, the yellow colour shows intermediate values, and the red colour shows large values. Figure 1 shows the overall estimated EIG for different subpopulations in Belgium. Figure 2 shows the overall estimated EIG for BMI population groups with underweight, normal weight, overweight, obesity, and severe obesity for all subpopulations. For example, Fig. 1 shows that on average Belgian males in 1997 consumed about 13 kcal/day more than their equilibrium energy expenditure while Belgian females consumed about 19 kcal/day more than their equilibrium energy expenditure. Figure 2 shows that on average males with severe obesity in Belgium in 1997 consumed about 22 kcal/day more than their equilibrium energy expenditure, while for females this figure was 36 kcal/day.

### EIG for the belgian men and women across BMI groups

As shown in Fig. 1, the overall EIG for Belgian men showed a slight drop until 2008 and then thereafter steadily increased up to 2018 (last health survey conducted). For Belgian women, the overall EIG decreased until 2004 and then increased with a peak around 2013 and negative values in 2018. As Fig. 2 shows, all BMI groups among Belgian men showed a drop in their EIG over time until 2004 or 2008, with an increase afterwards. All the BMI groups showed positive values for their EIG in 2018.

Moreover, men with obesity and severe obesity showed a larger value for their EIG compared to men with normal weight and overweight. Underweight men also showed a slightly larger EIG compared to normal weight men. Among Belgian women, EIG decreased over time across all weight groups until 2004 and then increased with a peak around 2013.

All the BMI groups among women showed negative EIGs in 2018. The magnitude of EIG was larger for heavier BMI groups across all survey years except 2018.

### EIG for the flemish subpopulation across BMI groups

Among Flemish men, the overall EIG increased slightly over time with a peak around 2001 and then decreased until 2008 with a rapid increase afterwards (Fig. 1). The overall EIG for Flemish men was positive in 2018. For Flemish women, the overall EIG decreased significantly over time until around 2004 and then increased afterwards with a peak in 2013 (Fig. 1). The overall EIG showed negative values in the 2018 for Flemish women. As Fig. 2 shows, the EIG for Flemish men increased over time for all weight groups until 2001, followed by a drop afterwards until 2008 and then increased for all weight groups until 2018.

Flemish men with severe obesity showed higher values for their EIG in year 2004 afterward compared to the rest of the BMI groups, while this was not the case for Flemish men with obesity. Among Flemish women, all the BMI groups showed negative values for their EIGs in 2018.

### EIG for the Brussels subpopulation across BMI groups

As Fig. 1 shows, the overall EIG for Brussels men decreased over time until 2004 and then increased afterwards with a peak in 2013. The overall EIG was negative in 2018 for Brussels men. For Brussel’s women, the overall EIG decreased until 2004 and then increased afterwards with a peak in 2013. The overall EIG showed negative values in 2018 for Brussels women. As Fig. 2 shows, among Brussels men the EIG decreased over time for all BMI classes (except for sever obesity) until 2004 and then increased with a peak in 2013. The EIG for Brussels men with severe obesity decreased continuously over time from 1997 to 2018. All weight groups (except underweight population) showed negative values for EIG around 2018. For men with severe obesity the EIG decreased continuously over time with negative values in 2018. At 2018 all the BMI groups showed negative EIG except underweight men.

For Brussels women across all BMI groups the EIG decreased until 2004 and then increased afterwards with a peak in 2013. All the BMI groups showed negative EIG in 2018.

### EIG for the Walloon Subpopulation across BMI groups

The overall EIG for Walloon men decreased until 2004 and then steadily increased with a peak in 2013 (Fig. 1). For Walloon women, the overall EIG decreased over time until 2011 and then increased afterwards. The overall EIG was positive in 2018 for both Walloon men and women. The EIG for underweight and normal weight Walloon men was larger than the EIG for men with overweight and obesity across all years.

For Walloon women, the EIG of heavier BMI groups was larger than lighter BMI groups across all years. The EIG across all BMI groups among Walloon men and women was positive in 2018.

### MEG

**Fig. 3:**
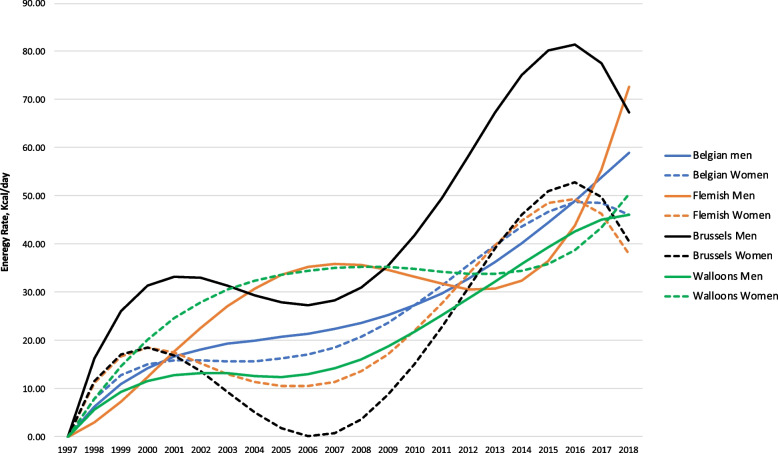
Estimated maintenance energy gap (MEG, Kcal/day) for different subpopulations among Belgian adults (rescaled to start from zero)

The estimated MEG (increased energy intake needed to maintain higher average body weights compared with an initial distribution of body weight in 1997) also quantifies the public health challenges to reverse the obesity prevalence to that of the late 1990s. The magnitude of the MEG for different subpopulation compared with that of 1997 (the first year for which data were available) is shown in Fig. 3. Comparing the BMI distribution in 2018 with that of 1997, the MEG measures the change that is needed to reverse the obesity trends. The MEG for Belgian men was estimated to be about 59 kcal/d in 2018. This means that Belgian men consumed (and expended) on average 59 kcal/d more in 2018 than the energy they were consuming (and expending) in 1997 to maintain their heavier body weight. There were some differences for males by region. The MEG for Flemish males was about 73 kcal/day in 2018 which is more than double the MEG in 2011. The MEG for Brussels’ males was about 67 kcal/day in 2018 while for Walloon males it was about 46 kcal/day.

The MEG for Belgian women was 46 kcal/day in 2018 which is triple the MEG in 2003. Also, for females there were some differences by region. The MEG in 2018 was about 38 kcal/day for Flemish women, 41 kcal/day for Brussels’ women, and 51 kcal/day for Walloon women.

## Discussion

In this paper, we quantified the dynamics of the EIG among Belgian adults over the past two decades.

The EIG captures the average daily difference between energy intake and expenditure that manages the speed of change in body weight. Our results showed considerable heterogeneity in the dynamics of EIG changes across the different subpopulations defined based on their gender, Belgian regions, and BMI category.

In Flanders, the EIG among men showed a substantial increase over the last five years (2013–2018) after a decreasing trend from 2001 to 2008. Among Flemish females the EIG increased since 2004 and then substantially decreased over the last five years (2013–2018) becoming negative around 2018. In Brussels the EIG substantially decreased and became negative for both males and females over the period 2013–2018. For Wallonia EIG substantially decreased over the past five years for males while considerably increased for females over the period 2013–2018. EIG was positive in 2018 for both Walloon males and females. Based on the observed trends in estimated EIG, the increase in overweight and obesity prevalence accelerated for Flemish males and Walloon females, decelerated for Walloon males, while in Brussels and for Flemish females the overweight and obesity rates started to decrease in 2018. According to the system dynamics model, subpopulations continue to gain weight until their average EIG is zero; only then will the obesity epidemic abate. Furthermore, a real decline in overweight and obesity will need negative EIG values among all subpopulations which is not yet the case in Belgium, only for some subpopulations since the last survey in 2018.

There was some heterogeneity in the patterns by BMI class so that subpopulations at different points in time could have strong gradients, with a higher BMI being associated with a higher or a lower EIG, or other patterns of gradients. No consistent patterns emerged over time. It has been proposed that people with high BMI are more likely to gain more weight in the future compared with those with lower BMI because of several feedback cycles whereby the mechanical, medical, dieting, psychological, and social consequences of obesity promote further weight gain [24]. Although the common right-skewed distribution of BMI may still in part be due to these feedback cycles whereby obesity begets obesity, this study did not provide consistent evidence to support this contention.

For the period 2013–2018, the MEG is much higher in 2018 than in 2013 for Belgian males and females. Across subpopulations, the same behavior was observed for Walloons males and females, as well as Flemish males. For Flemish females and Brussels males and females the MEG increased since 2013 with a peak around 2016. In 2018 the MEG for these subpopulations was similar to that of 2013. These findings suggest that, overall, there has not been a reduction in energy intake among the Belgian population during the period of the Convenant balanced diets (2012–2017). Within the Convenant, the food industry committed to reduce population energy intake by 5% through reducing sugar and saturated fat in their products, between 2012 and 2017 [7]. This observation may imply the ineffectiveness of industry self-regulation to improve the healthiness of the food supply. It is widely recognized that strong government regulations are needed in order to prevent and reduce overweight and obesity, as well as a comprehensive approach including a combination of interventions, such as restrictions of unhealthy food marketing, front-of-pack labeling and economic interventions [25]. With respect to physical activity over period 2013–2018, the percentage of population 15 years and older at health risk due to a lack of exercise in leisure time remained fairly stable over time since 2004 (32.9% in 1997, 35.4% in 2001, 27.1% in 2004, 28.3% in 2008 and 27.6% in 2013), according to the HIS [26].

The study has several limitations. First, the use of BMI as an anthropometric index of overweight and obesity has been shown to introduce bias from misclassification.

Moreover, height and weight were self-reported in the HIS surveys which can lead to an underestimation of the population BMI. The Belgian Food Consumption Surveys (FCS) generally measure height and weight, but only 2 waves of data are available as these surveys are organized less regularly. Obesity prevalence has been compared for adults aged 18–64 years between the HIS (self-reported) and the FCS (measured) using the data from 2014 as conditions are comparable (same time span, target group and same method of sampling). The corrected obesity prevalence of the HIS calculated for the population aged 18–64 years was 17.2% (before correction this prevalence was 12.8%) and approaches the prevalence of the FCS which is 19,4%. This implies that the problem of obesity in Belgium is probably 4% points higher than the initial prevalence [27] and suggests an underestimation of the EIG and MEG.

The results of this study can help to predict the future trends in weight gain or loss and give the estimated past trajectories of the EIG, emphasizing the predictive value of this study.

Finally, this study helps us to detect the magnitude and direction of responses of each subpopulation to interventions, emphasizing the evaluative value of this study. Our model can also evaluate (or predict) the effect of different interventions impacting energy intake on the change in prevalence of obesity and overweight in a population.

## Conclusions

The detailed trends of the EIG and MEG illustrate and quantify the heterogeneity in energy imbalance responsible for obesity trends by sex, region and BMI subpopulations in Belgium. The increase in overweight and obesity prevalence accelerated for Flemish males and Walloon females, decelerated for Walloon males, while in Brussels and for Flemish females the overweight and obesity rates started to decrease in 2018.

These findings help describe the patterns in obesity trends and suggest the need for customizing targets/policy interventions for different subpopulations.

## Supplementary Information

**Additional file 1.**

## Data Availability

The datasets generated and analysed during the current study are not publicly available due to the fact that these data fall under the Act of 30 July 2018 on the protection of privacy in relation to the processing of personal data. Therefore, these data can only be made available to third parties after approval of an authorization application by the Social Security and Health Chamber of the Information Security Committee. The datasets are available from the corresponding author on request according to certain conditions and fees. The procedure to obtain access (in English, Dutch or French) can be found on https://www.sciensano.be/en/node/55737/health-interview-survey-microdata-request-procedure.

## Abbreviations

BMI: Body Mass Index
EIG: Energy imbalance gap
HIS: Health Interview Survey
MEG: Maintenance energy gap
