# Supplementary material for: Trends in adults’ energy imbalance gaps over two decades in Belgium using system dynamics modelling
Source: BMC Nutr. 2023 May 27;9:66. doi: 10.1186/s40795-023-00721-0 (PMC10223905; doi:10.1186/s40795-023-00721-0)
Supplement: Supplementary file 1 — Additional file 1. [file 40795_2023_721_MOESM1_ESM.pdf]

Figures S1: Estimated overall energy imbalance gap (EIG) in kilocalories per day for Belgian adults by gender and region across all years

	Gender	1997	1998	1999	2000	2001	2002	2003	2004	2005	2006	2007	2008	2009	2010	2011	2012	2013	2014	2015	2016	2017	2018
Belgian	Males	12.7	11.0	9.6	8.4	7.4	6.6	6.1	5.7	5.5	5.4	5.4	5.6	5.9	6.3	6.8	7.3	7.9	8.5	9.1	9.7	10.4	11.0
	Females	19.1	13.9	9.8	6.7	4.5	3.2	2.6	2.7	3.1	4.0	5.1	6.3	7.5	8.6	9.4	9.9	9.9	9.2	7.8	5.6	2.3	-2.0
Flemish	Males	5.0	7.7	9.4	10.3	10.5	10.1	9.3	8.1	6.7	5.3	3.9	2.6	1.7	1.1	1.1	1.8	3.3	5.7	9.2	14.0	20.2	28.1
	Females	28.7	19.1	11.6	6.2	2.5	0.4	-0.5	-0.2	0.9	2.6	4.7	7.0	9.3	11.3	12.8	13.5	13.3	11.8	8.9	4.3	-2.2	-10.6
Brussels	Males	29.0	20.7	14.1	9.0	5.4	3.1	2.0	1.9	2.6	3.8	5.4	7.3	9.1	10.7	11.9	12.4	12.1	10.8	8.3	4.5	-0.8	-7.6
	Females	30.1	19.3	10.9	4.8	0.8	-1.5	-2.3	-1.8	-0.4	1.7	4.3	7.2	10.0	12.5	14.4	15.5	15.5	14.0	10.9	5.9	-1.4	-10.9
Walloons	Males	12.5	10.3	8.7	7.5	6.8	6.5	6.5	6.7	7.1	7.7	8.4	9.0	9.6	10.1	10.5	10.6	10.4	9.9	8.9	7.5	5.6	3.1
	Females	12.6	12.0	11.2	10.2	9.1	8.0	6.8	5.7	4.6	3.5	2.6	1.9	1.4	1.1	1.0	1.3	2.0	3.0	4.5	6.4	8.9	12.0

Figure S2: Estimated energy imbalance gap (EIG) in kilocalories per day for Belgian adults (age 20-74 years) by gender, region, and weight group in Belgium across all years

	Gender	Weight Group	1997	1998	1999	2000	2001	2002	2003	2004	2005	2006	2007	2008	2009	2010	2011	2012	2013	2014	2015	2016	2017	2018
Begian	Male	Under Weight	14.3	13.0	11.9	10.9	10.2	9.6	9.1	8.9	8.7	8.7	8.8	9.0	9.2	9.6	10.0	10.5	11.0	11.5	12.1	12.7	13.2	13.8
		Normal Weight	12.2	10.6	9.3	8.2	7.3	6.6	6.1	5.8	5.6	5.6	5.6	5.8	6.1	6.5	6.9	7.4	8.0	8.6	9.2	9.8	10.4	11.0
		Over Weight	12.2	10.4	8.8	7.5	6.5	5.7	5.1	4.6	4.4	4.3	4.3	4.5	4.8	5.2	5.6	6.2	6.8	7.4	8.0	8.7	9.3	10.0
		Obese	14.7	12.7	10.9	9.5	8.3	7.3	6.6	6.1	5.7	5.6	5.6	5.7	6.0	6.4	6.9	7.4	8.0	8.7	9.4	10.0	10.7	11.4
		Severly Obese	22.0	19.9	18.0	16.5	15.2	14.1	13.4	12.8	12.5	12.4	12.4	12.6	12.9	13.4	13.9	14.6	15.3	16.0	16.7	17.5	18.2	18.9
	Female	Under Weight	14.9	10.5	7.1	4.7	3.0	2.0	1.7	1.8	2.3	3.1	4.2	5.3	6.4	7.4	8.2	8.7	8.8	8.4	7.4	5.7	3.2	-0.2
		Normal Weight	17.3	12.2	8.3	5.4	3.4	2.2	1.7	1.8	2.3	3.1	4.2	5.4	6.6	7.6	8.5	8.9	9.0	8.4	7.2	5.2	2.2	-1.7
		Over Weight	21.2	15.3	10.7	7.3	5.0	3.5	2.8	2.8	3.2	4.1	5.2	6.4	7.6	8.7	9.5	10.0	9.9	9.2	7.6	5.2	1.8	-2.8
		Obese	27.0	20.1	14.8	10.8	8.0	6.2	5.3	5.0	5.4	6.2	7.3	8.5	9.8	10.8	11.6	12.0	11.7	10.8	8.9	6.1	2.0	-3.3
		Severly Obese	35.6	27.9	21.8	17.3	14.0	11.8	10.7	10.4	10.8	11.6	12.7	14.0	15.2	16.3	17.0	17.2	16.7	15.4	13.0	9.4	4.5	-1.9
Flemish	Male	Under Weight	11.4	13.2	14.3	14.6	14.4	13.7	12.6	11.3	9.8	8.3	6.7	5.3	4.1	3.3	2.9	3.0	3.8	5.4	7.7	11.1	15.5	21.1
		Normal Weight	6.9	9.2	10.7	11.4	11.4	10.9	9.9	8.6	7.2	5.6	4.1	2.8	1.7	1.0	0.8	1.2	2.3	4.3	7.3	11.4	16.7	23.4
		Over Weight	3.1	6.1	8.0	9.1	9.4	9.1	8.3	7.2	5.9	4.4	3.0	1.8	0.9	0.4	0.5	1.2	2.8	5.4	9.1	14.0	20.3	28.2
		Obese	0.5	4.2	6.7	8.3	9.0	9.1	8.6	7.7	6.5	5.3	4.1	3.1	2.5	2.3	2.8	4.0	6.2	9.4	13.9	19.8	27.3	36.5
		Severly Obese	0.2	5.0	8.5	10.9	12.3	13.0	13.0	12.7	12.0	11.2	10.5	10.1	10.0	10.5	11.8	14.0	17.2	21.6	27.5	34.9	44.1	55.4
	Female	Under Weight	25.8	17.7	11.6	7.2	4.2	2.5	1.9	2.2	3.2	4.8	6.6	8.6	10.6	12.4	13.7	14.4	14.4	13.3	11.0	7.4	2.2	-4.7
		Normal Weight	26.7	17.5	10.4	5.3	1.8	-0.2	-0.9	-0.6	0.5	2.1	4.2	6.4	8.6	10.5	12.0	12.7	12.5	11.2	8.5	4.3	-1.7	-9.5
		Over Weight	30.0	19.5	11.4	5.5	1.5	-0.9	-1.8	-1.6	-0.5	1.3	3.5	5.9	8.3	10.4	11.9	12.6	12.3	10.7	7.6	2.7	-4.1	-13.2
		Obese	37.0	24.8	15.4	8.5	3.9	1.1	-0.1	-0.1	1.0	2.9	5.3	7.9	10.4	12.6	14.2	14.9	14.4	12.5	8.8	3.2	-4.6	-14.9
		Severly Obese	48.3	34.7	24.3	16.6	11.3	8.1	6.7	6.8	8.0	10.1	12.8	15.7	18.6	21.1	22.9	23.6	23.0	20.6	16.3	9.6	0.3	-11.9
Brussels	Male	Under Weight	19.7	13.3	8.5	5.2	3.1	2.1	2.1	2.8	4.2	6.0	8.2	10.6	13.0	15.2	17.2	18.6	19.4	19.5	18.6	16.7	13.7	9.3
		Normal Weight	24.4	16.3	10.1	5.7	2.6	0.9	0.3	0.6	1.6	3.2	5.2	7.3	9.5	11.4	13.1	14.2	14.5	13.9	12.3	9.4	5.0	-1.0
		Over Weight	32.8	23.1	15.5	9.7	5.6	2.9	1.5	1.1	1.6	2.6	4.1	5.8	7.5	9.0	10.1	10.6	10.3	8.9	6.3	2.3	-3.3	-10.7
		Obese	47.0	35.3	25.8	18.4	12.9	9.0	6.5	5.2	4.8	5.1	5.9	6.9	7.9	8.7	9.1	8.7	7.5	5.1	1.3	-4.0	-11.2	-20.2
		Severly Obese	71.1	57.7	46.6	37.6	30.6	25.2	21.3	18.6	16.9	15.9	15.4	15.1	14.7	14.0	12.8	10.7	7.6	3.2	-2.7	-10.5	-20.2	-32.2
	Female	Under Weight	25.6	16.4	9.4	4.4	1.1	-0.7	-1.3	-0.8	0.5	2.4	4.6	7.0	9.4	11.5	13.2	14.1	14.1	13.0	10.5	6.4	0.6	-7.2
		Normal Weight	28.0	17.5	9.6	3.8	0.0	-2.1	-2.8	-2.3	-1.0	1.1	3.6	6.3	9.0	11.3	13.2	14.2	14.1	12.8	9.9	5.2	-1.4	-10.2
		Over Weight	32.4	20.4	11.2	4.6	0.2	-2.3	-3.1	-2.7	-1.2	1.1	3.9	6.9	9.9	12.5	14.5	15.6	15.4	13.9	10.5	5.1	-2.5	-12.6
		Obese	38.9	25.2	14.8	7.2	2.2	-0.7	-1.8	-1.3	0.3	2.8	5.8	9.1	12.4	15.2	17.4	18.5	18.3	16.4	12.6	6.4	-2.2	-13.7
		Severly Obese	47.5	32.8	21.4	13.0	7.3	4.0	2.8	3.2	5.0	7.8	11.2	15.1	18.8	22.2	24.8	26.2	26.0	23.9	19.4	12.2	2.0	-11.5
Walloons	Male	Under Weight	17.6	16.0	14.9	14.1	13.6	13.3	13.3	13.6	14.0	14.6	15.2	15.8	16.3	16.8	17.2	17.3	17.2	16.9	16.2	15.2	13.7	11.8
		Normal Weight	12.5	10.6	9.1	8.1	7.5	7.2	7.2	7.5	8.0	8.5	9.1	9.8	10.4	10.9	11.2	11.4	11.3	10.9	10.1	8.9	7.3	5.1
		Over Weight	10.9	8.6	6.9	5.7	4.9	4.5	4.5	4.7	5.1	5.7	6.3	6.9	7.5	8.0	8.4	8.5	8.3	7.7	6.8	5.4	3.4	0.9
		Obese	13.4	10.8	8.8	7.4	6.4	5.9	5.8	6.0	6.3	6.9	7.5	8.1	8.7	9.2	9.5	9.5	9.3	8.6	7.4	5.8	3.5	0.6
		Severly Obese	21.4	18.8	16.9	15.5	14.7	14.4	14.4	14.8	15.4	16.2	17.0	17.9	18.7	19.4	19.9	20.1	19.9	19.2	17.9	15.9	13.2	9.7
	Female	Under Weight	9.2	8.6	7.9	7.0	6.0	5.0	4.0	3.0	2.0	1.1	0.3	-0.3	-0.8	-1.1	-1.2	-1.0	-0.5	0.3	1.4	3.0	4.9	7.3
		Normal Weight	11.7	11.0	10.1	9.2	8.1	7.0	5.9	4.8	3.7	2.7	1.9	1.2	0.7	0.4	0.3	0.6	1.1	2.1	3.4	5.2	7.4	10.1
		Over Weight	14.2	13.5	12.6	11.5	10.4	9.2	7.9	6.7	5.5	4.4	3.5	2.7	2.1	1.8	1.8	2.1	2.8	3.9	5.5	7.5	10.1	13.3
		Obese	16.6	15.8	14.8	13.6	12.4	11.0	9.7	8.3	7.1	5.9	4.9	4.0	3.5	3.2	3.2	3.6	4.4	5.7	7.5	9.8	12.8	16.4
		Severly Obese	18.9	18.0	17.0	15.7	14.3	12.9	11.4	10.0	8.6	7.3	6.3	5.4	4.8	4.5	4.6	5.2	6.2	7.8	9.9	12.7	16.2	20.5
